# Supplementary material for: Scavenger receptor CD163 multimerises to allow uptake of diverse ligands
Source: Nat Commun. 2025 Jul 18;16:6623. doi: 10.1038/s41467-025-62054-9 (PMC12274614; doi:10.1038/s41467-025-62054-9)
Supplement: Supplementary file 1 — Supplementary Information [file 41467_2025_62054_MOESM1_ESM.pdf]

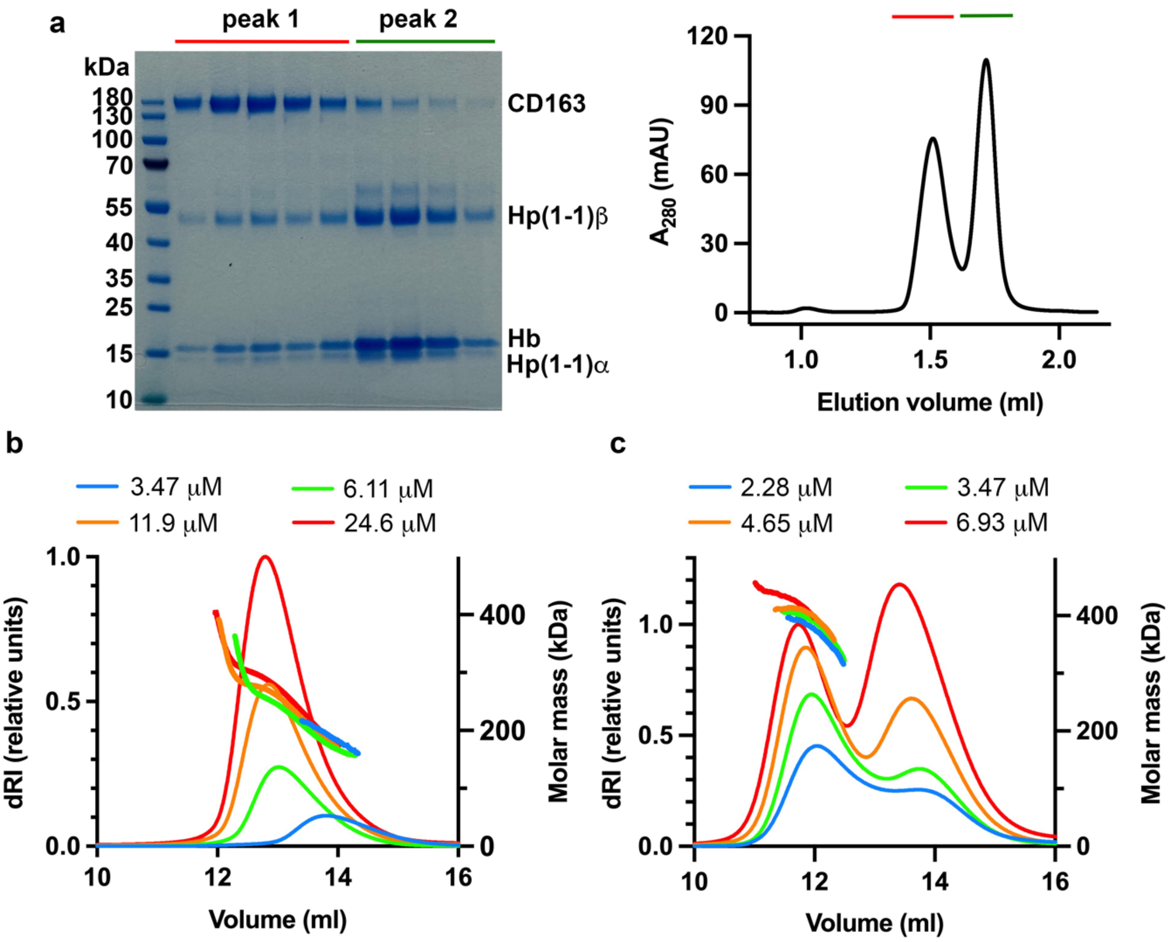

Supplementary Figure 1: Purification and characterisation of the CD163:HpHb complex.

a. Purification of a complex of CD163 and Hp(1-1)Hb. The right-hand panel shows the size-exclusion profile on a Superose 6 3.2/300 column while the left-hand panel shows a Coomassie-stained gel for the fractions, with peak 1 containing the liganded CD163 complex. **b.** SEC-MALLS data for unliganded CD163 at four different CD163 concentrations. The left y-axis shows the differential refractive index, while the right y-axis depicts the molecular weight. This is representative of 2 repeats. **c.** SEC-MALLS data for a complex of CD163 and Hp(1-1)Hb at four different CD163 concentrations and a constant HpHb concentration of 1.18 μM Hp(1-1)Hb. This is representative of 2 repeats. For reference, the predicted molecular weights are ~ 480 kDa and 370 kDa for the trimeric and dimeric liganded complex, respectively.

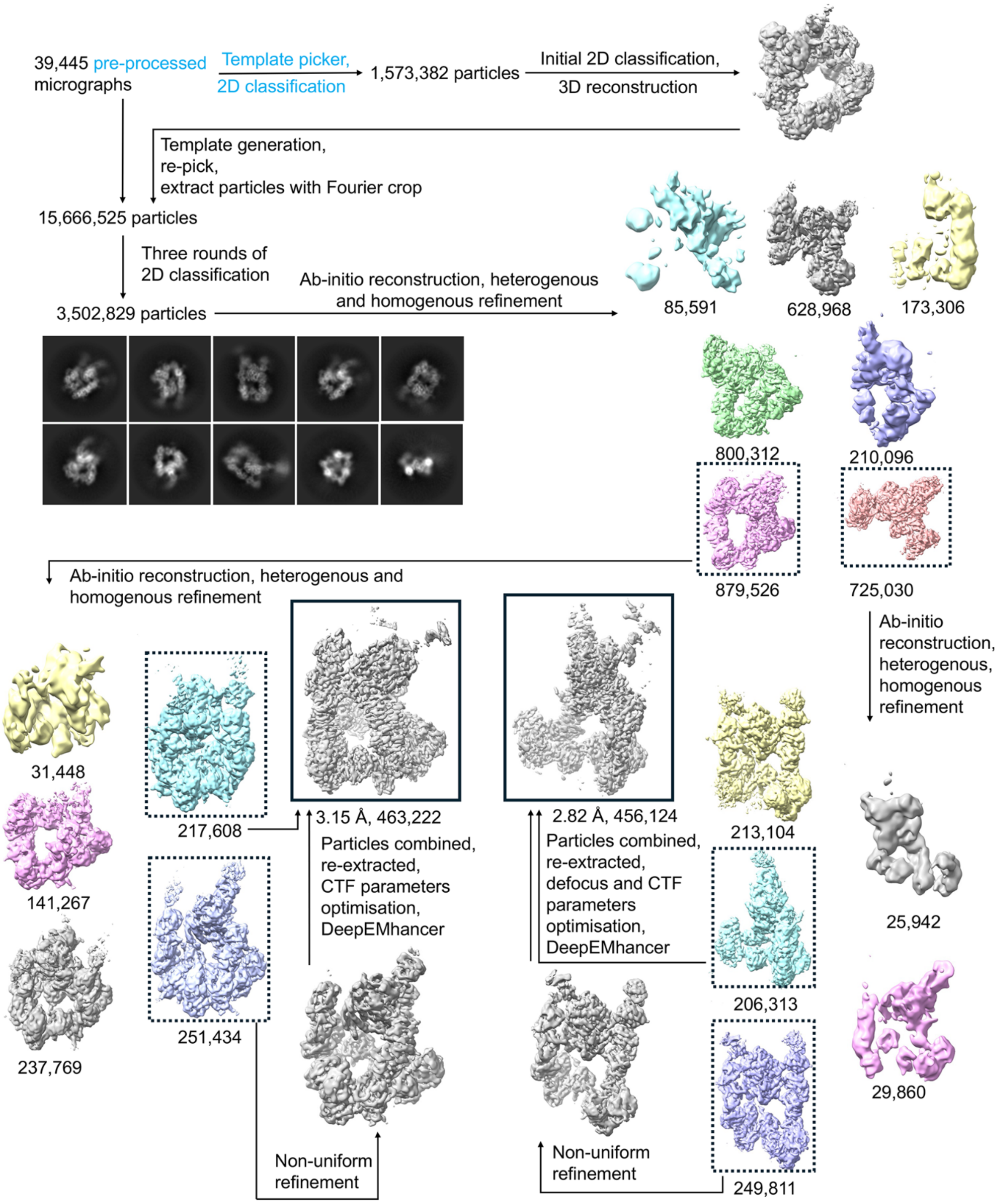

Supplementary Figure 2: Cryo-EM image processing scheme for HpHb-bound CD163.

Steps that were performed in SIMPLE are in blue whereas steps that were conducted in CryoSPARC are in black.

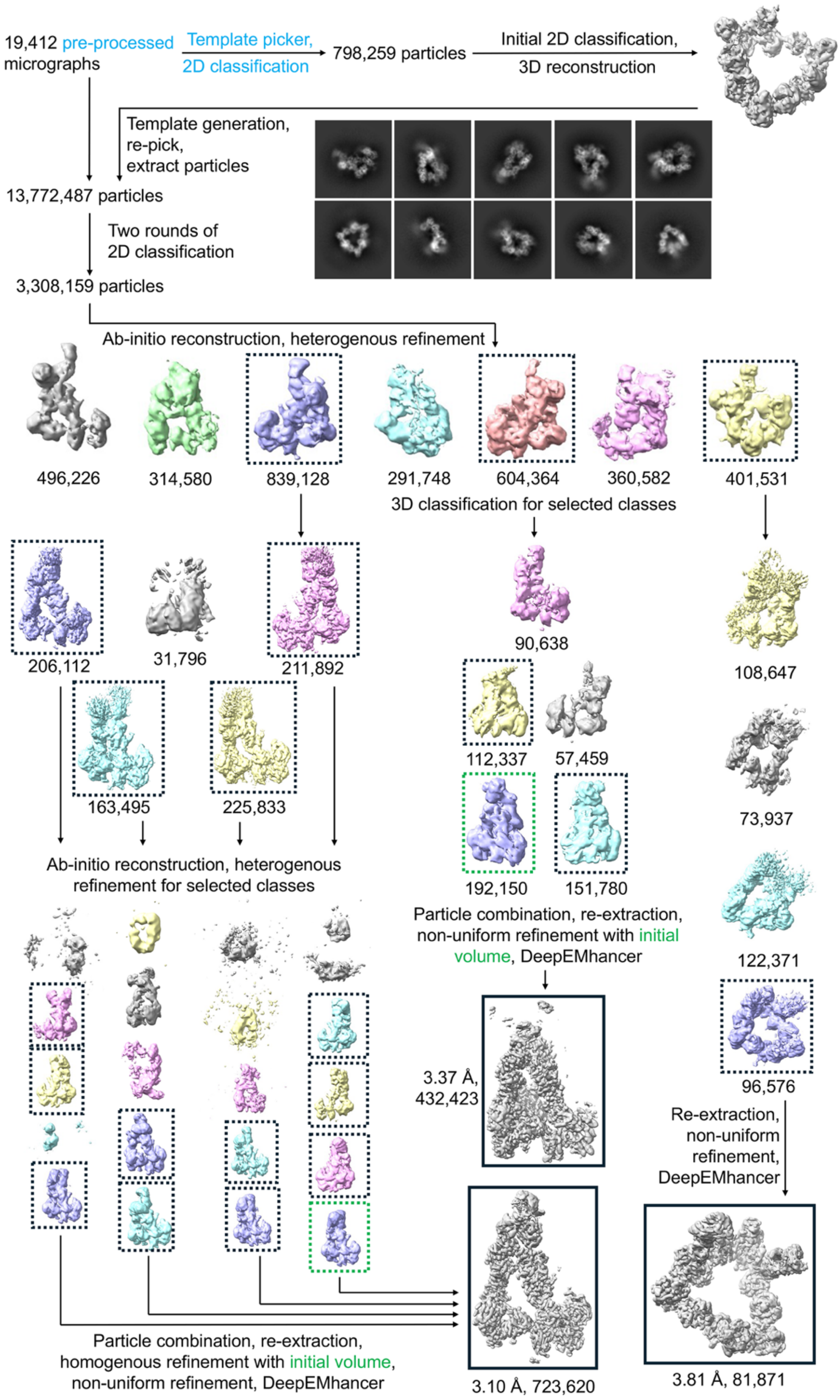

Supplementary Figure 3: Cryo-EM image processing scheme for unliganded CD163

Steps that were performed in SIMPLE are in blue whereas steps that were conducted in CryoSPARC are in black.

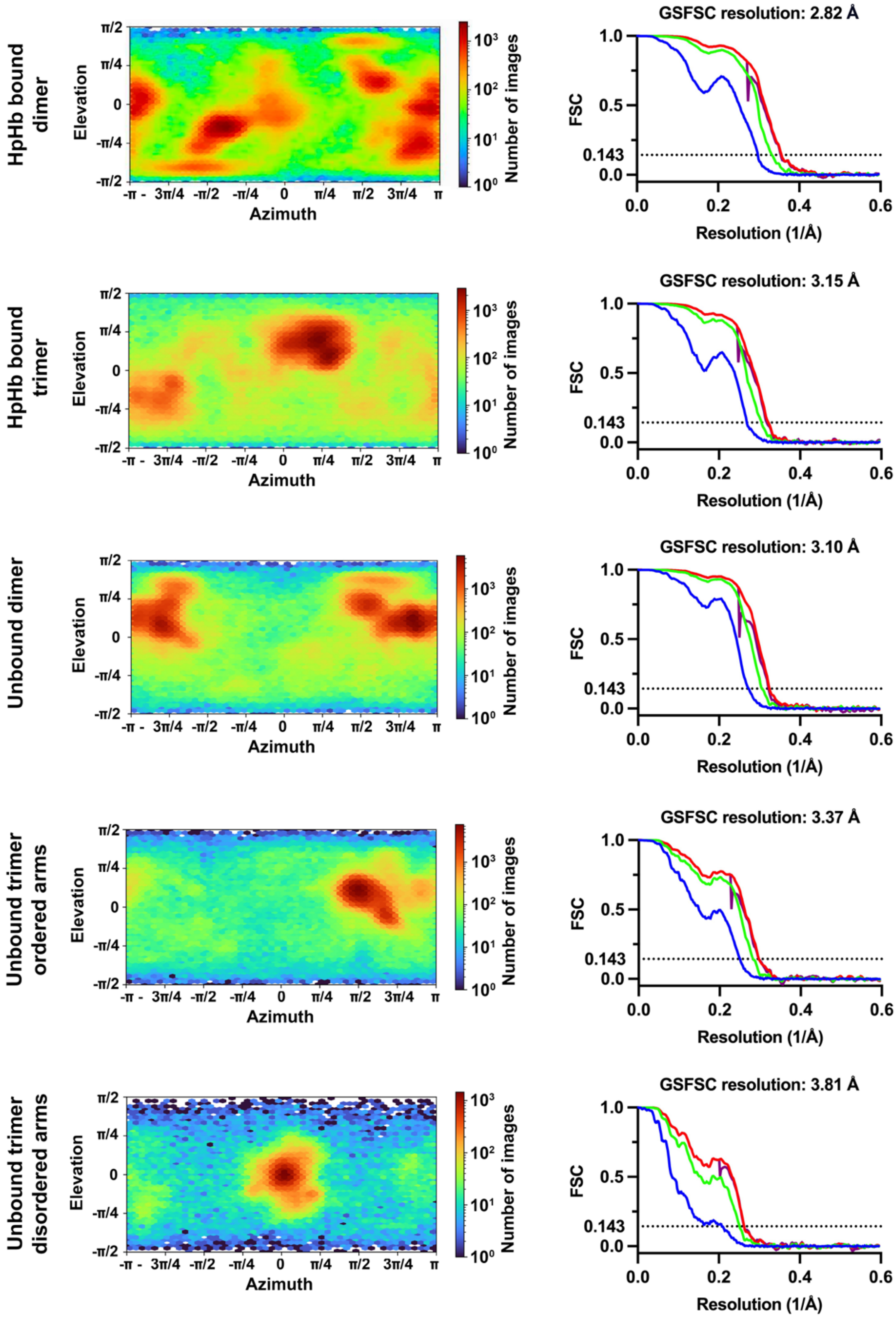

Supplementary Figure 4: Particle-view distributions and resolutions of reconstructions.

The left-hand panels show particle-view distributions for each of the five structures, while the right-hand panels display GSFSC curves. The blue, green, red and purple traces correspond to no mask, loose solvent mask, tight solvent mask and correlated calculations, respectively.

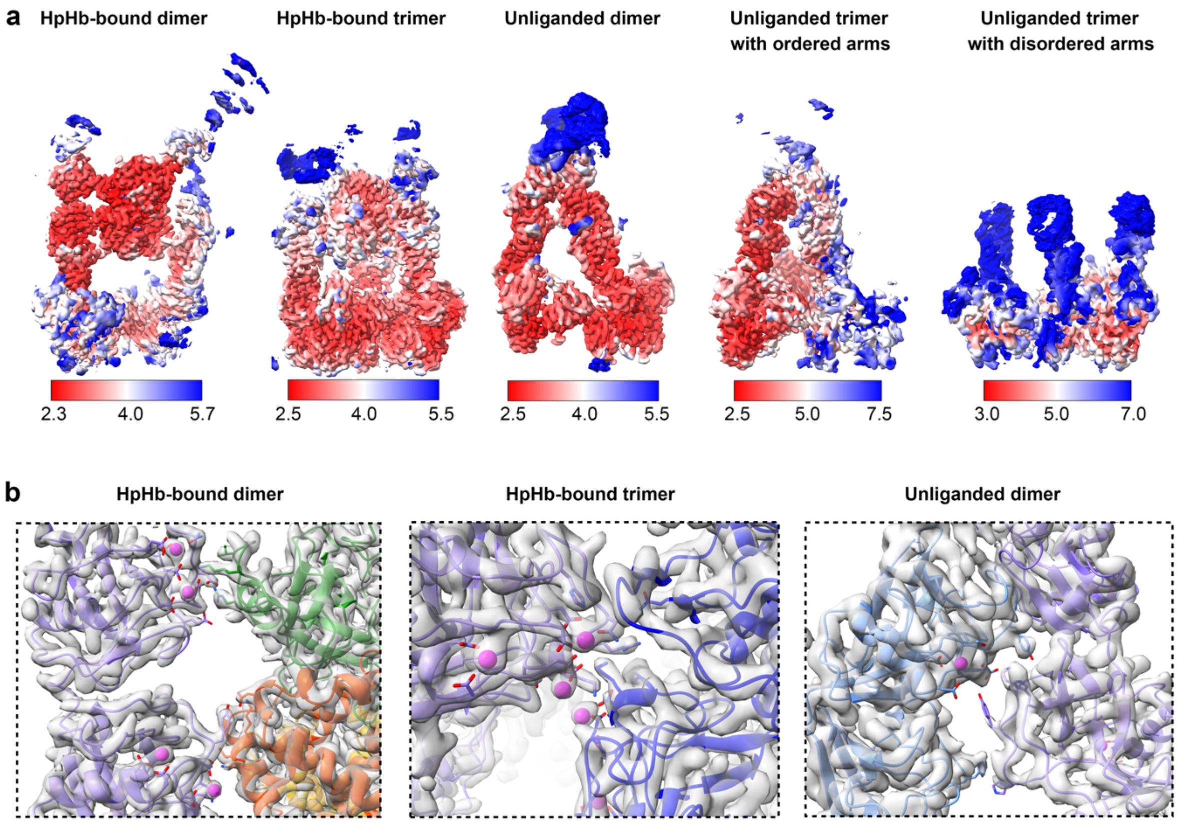

Supplementary Figure 5: Local resolution estimations and key structural features of liganded and unliganded CD163 complexes.

a. Maps derived from cryogenic electron microscopy, coloured according to local resolution. Below each panel is a key, with resolution values in Å. **b.** Close-up views of density maps and fitted models. The left-hand panel shows part of the interface between HpHb and the CD163 dimer. The central panel shows part of the interface between $CD163_A$ and $CD163_C$ in the trimeric complex. The right-hand panel shows part of the interface between the arms of unliganded CD163.

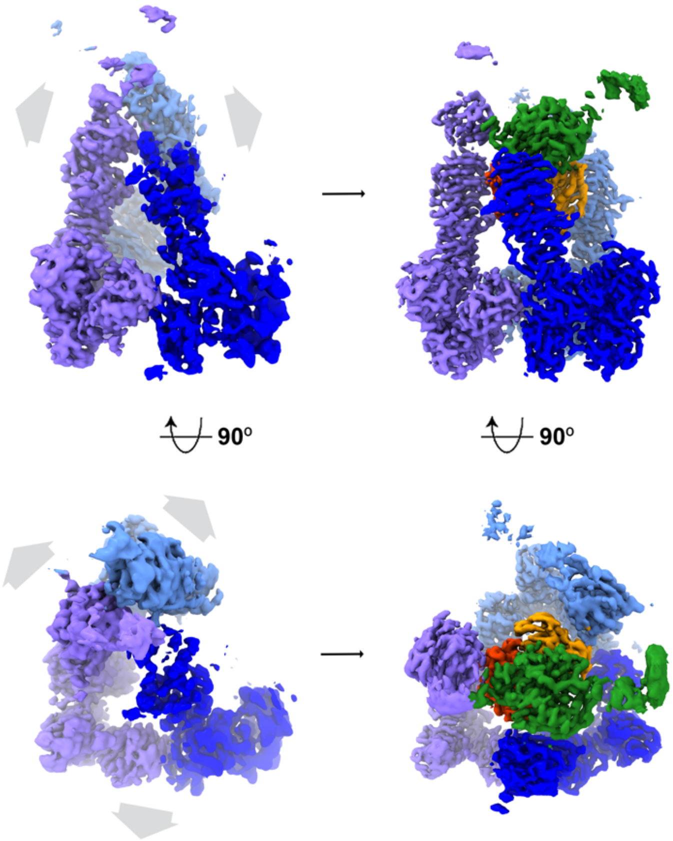

Supplementary Figure 6: A trimeric unliganded CD163 with arm-arm interactions.

The structure of a trimer of CD163 in the absence of ligand (left) and in the presence of HpHb (right), showing arm-arm contacts between the CD163 arms in the unliganded trimer, with the arms moving outwards to allow ligand binding.

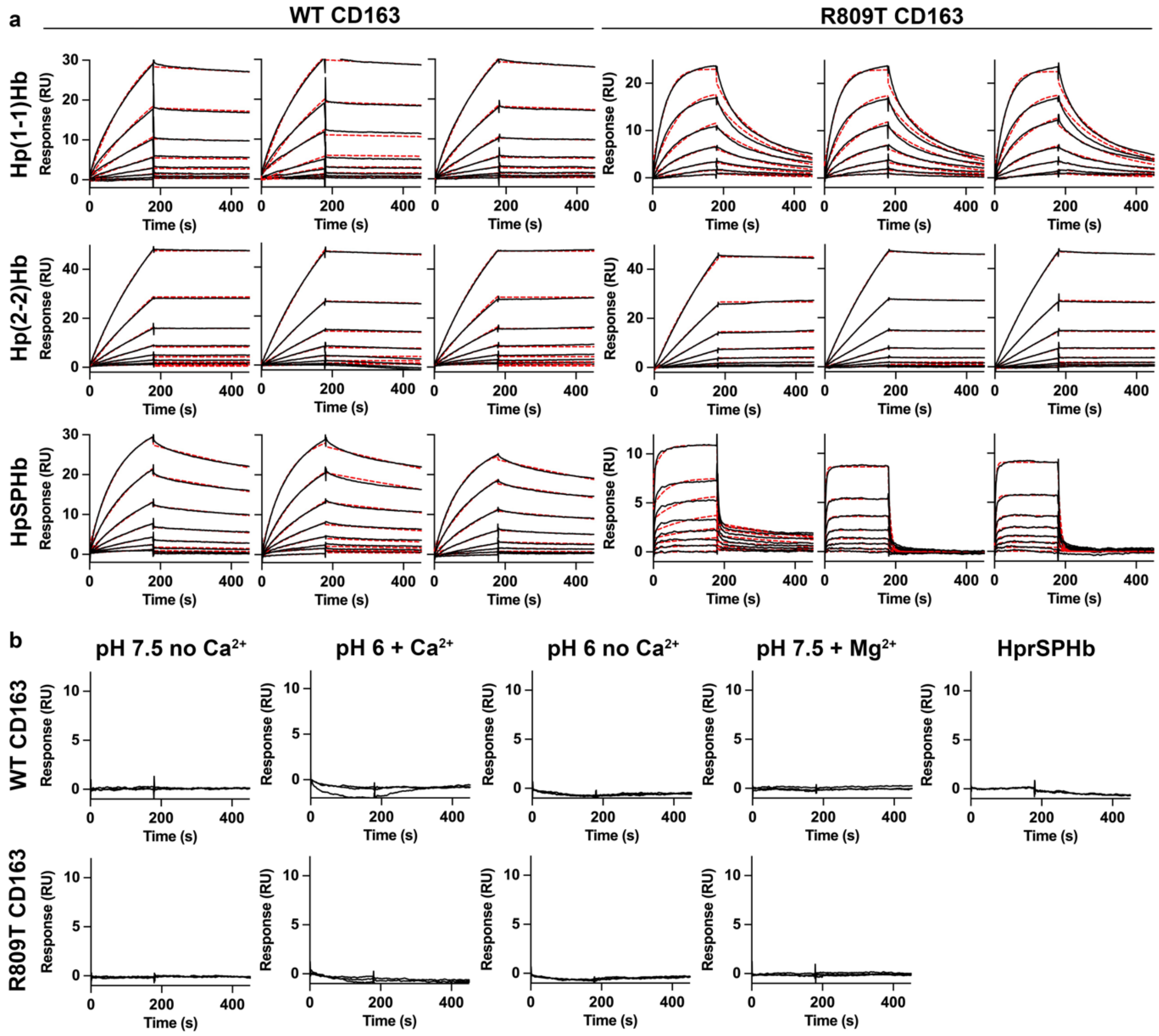

Supplementary Figure 7: SPR analysis of ligand binding to immobilised CD163 ectodomain.

a. SPR measurements of ligand binding. When WT CD163 was immobilised, two-fold dilution series were conducted from top concentrations of 10 nM for Hp(1-1)Hb, 10 nM for Hp(2-2)Hb and 20 nM for HpSPHb. When R809T CD163 was captured, two-fold dilution series from 5 nM Hp(1-1)Hb, 2.5 nM Hp(2-2)Hb and 80 nM HpSPHb were flowed over the chip surface. The graphs shown are the three technical replicates. **b.** SPR results in the absence of ligand binding. Three replicates are shown on every graph. The right-hand graph shows injections of HprSPHb at a concentration of 20 nM on a surface coated with WT CD163. The remaining curves show the effect of 10 nM Hp(1-1)Hb flowed over a surface coated with WT or R809T CD163, in one of four buffers: 20 mM HEPES pH 7.5, 150 mM NaCl (pH 7.5 no Ca^{2+}); 20 mM MES pH 6.0, 150 mM NaCl, 2.5 mM $CaCl_2$ (pH 6 + Ca^{2+}); 20 mM MES pH 6.0, 150 mM NaCl (pH 6 no Ca^{2+}) or 20 mM HEPES pH 7.5, 150 mM NaCl, 2.5 mM $MgCl_2$ (pH 7.5 + Mg^{2+}).

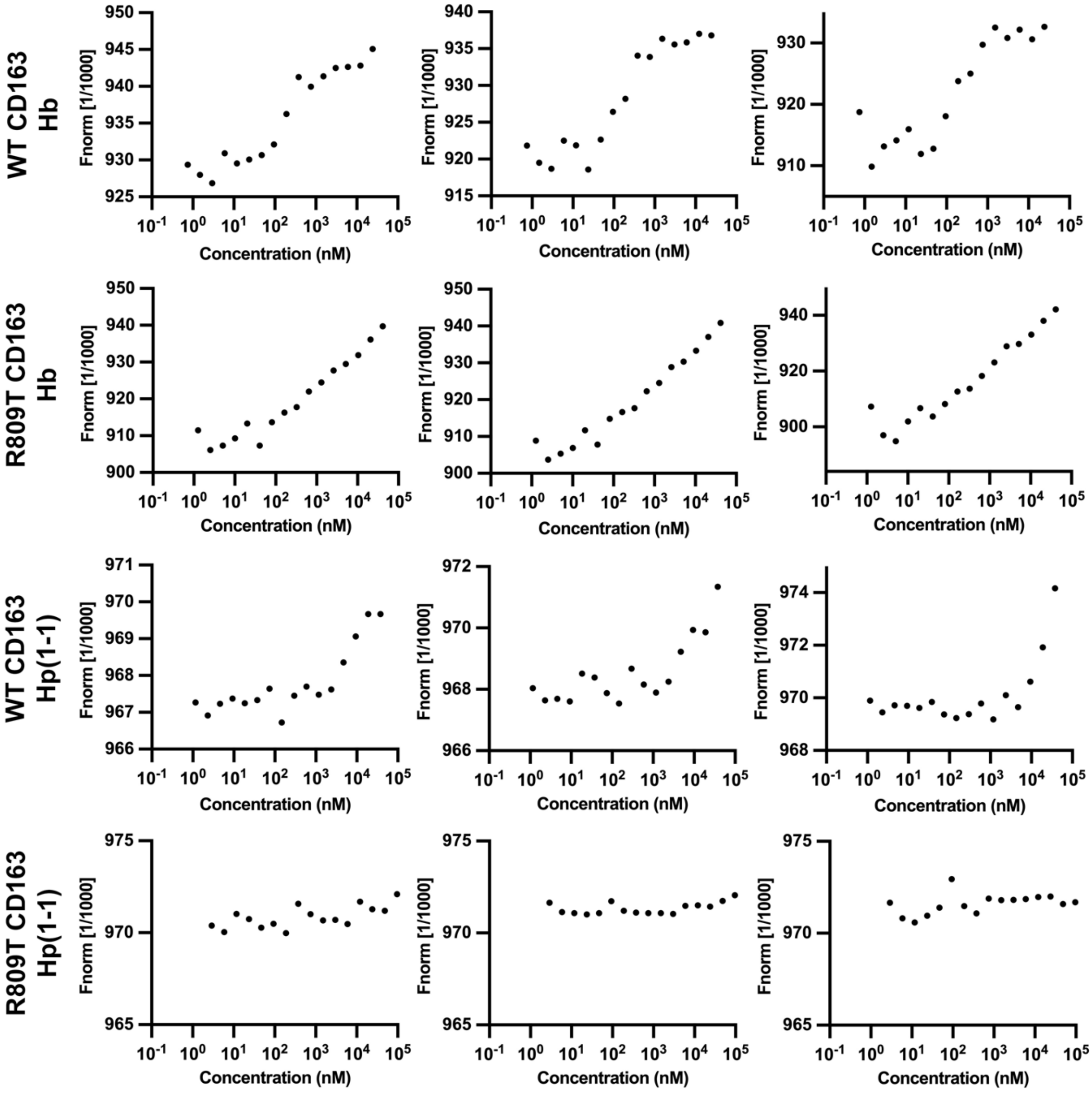

Supplementary Figure 8: Microscale thermophoresis.

MST analysis of the binding of Hp and Hb to WT CD163 or to the R809T mutant. To assess the interactions with Hb, 200 nM fluorescent Hb was mixed with two-fold dilution series of WT or R809T CD163 starting from 24.4 μM and 41.5 μM, respectively. To probe binding to Hp, 0.50 and 1.0 μM fluorescent Hp(1-1) were incubated with WT and R809T CD163 from 37.9 and 96.3 μM, respectively. Samples were measured in technical triplicates.

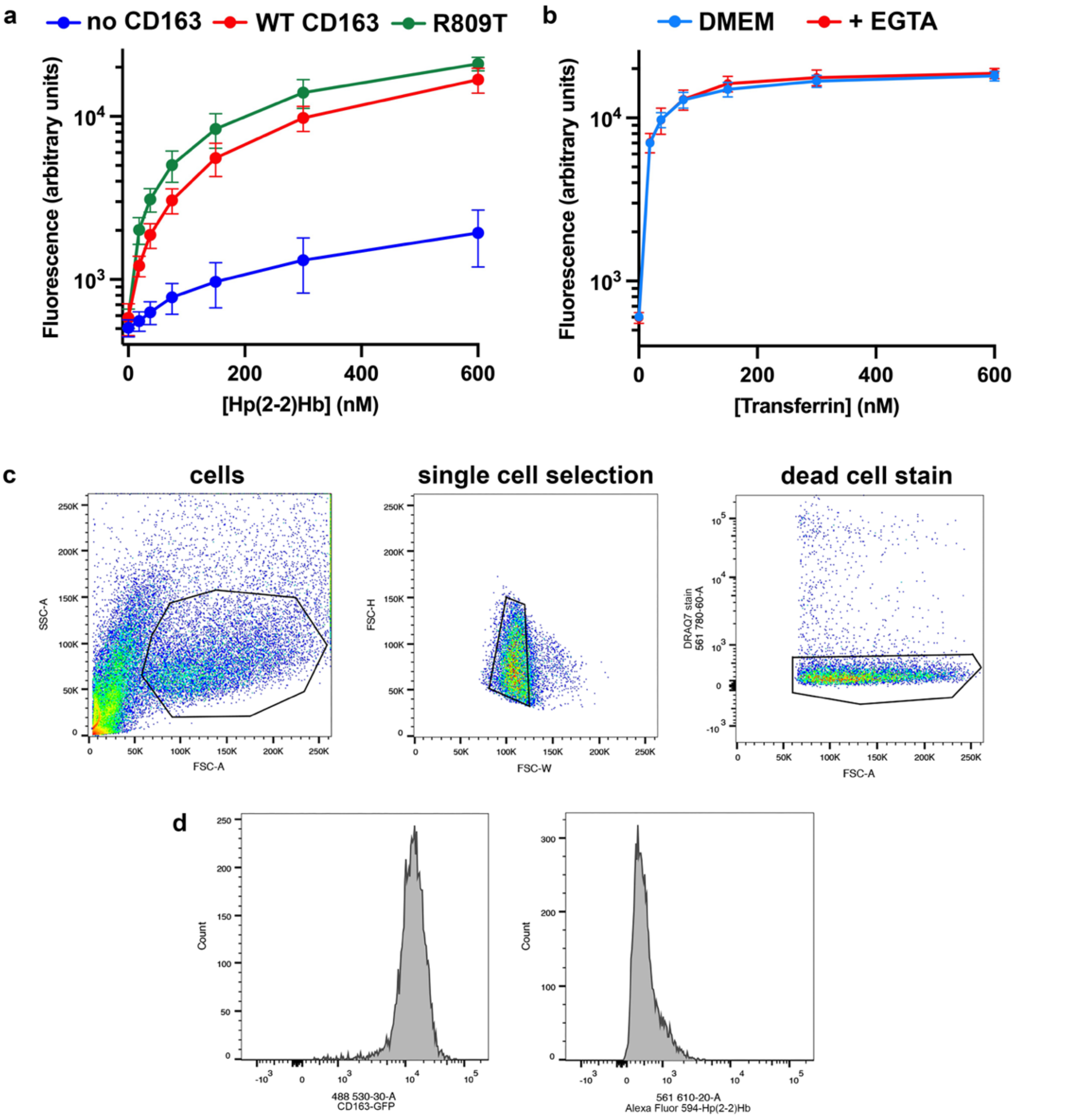

Supplementary Figure 9: Ligand uptake analysis using fluorescence-activated cell sorting.

a. Measurement of the uptake of fluorescently labelled Hp(2-2)Hb into untransfected HEK293 cells (blue) or into cells transfected with wild-type CD163 (red) or the R809T mutant (green). Each point represents the mean of three replicates and the error bars depict standard deviations. **b.** Measurement of the uptake of fluorescently labelled transferrin into HEK293 cells in standard DMEM media, or in DMEM with the addition of 2 mM EGTA to chelate all calcium ions. Each point represents the mean of three replicates and the error bars depict standard deviations. **c.** Gating scheme for the selection of live, single cells. The depicted gates were used for every sample in this study. **d.** Example of FACS histograms for HEK293 cells transfected with WT CD163 fused to GFP in the absence of ligand uptake. The gating strategy shown in c. was applied to obtain these histograms. CD163 expression was monitored using the left-hand histogram while ligand uptake was quantified by measuring the mean population fluorescence in the right-hand histogram.

Supplementary Table 1: Cryo-EM data collection, refinement and validation statistics for Hp(1-1)Hb-bound CD163

	Dimeric CD163:Hp(1-1)Hb	Trimeric CD163:Hp(1-1)Hb
**Data collection and processing**		
Microscope	Titan Krios	
Detector	Gatan K3 with BioQuantum Imaging Filter	
Magnification	58,149	
Voltage (kV)	300	
Electron exposure (e-/Å^2)	41.68	
Defocus range (μm)	-0.4 to -2.0	
Pixel size (Å)	0.832	
Symmetry imposed	C1	
Initial particle images (no.)	15,666,525	
Final particle images (no.)	456,124	463,222
Map resolution (Å)	2.82	3.15
FSC threshold 0.143		
Map resolution range (Å)	2.25 - 35.8	2.55 – 27.8
FSC threshold 0.5		
**Refinement**		
Initial model used	AlphaFold2, 4X0L, 4WJG	
Model resolution (Å)	3.06	3.22
FSC threshold 0.5		
Model composition		
Non-hydrogen atoms	15177	22064
Protein residues	1963	2856
Ligands	21	40
B factors (Å^2)		
Protein	79.41	80.63
Ligands	74.31	100.28
R.m.s deviations		
Bond lengths (Å)	0.004	0.004
Bond angles (°)	0.740	0.756
Validation		
MolProbity score	1.99	1.68
Clashscore	7.34	7.55
Rotamer outliers (%)	1.92	0.56
Ramachandran plot		
Favoured (%)	94.7	96.1
Allowed (%)	5.3	3.9
Disallowed (%)	0.0	0.0
Model vs. data fit		
CC (mask)	0.78	0.78
CC (box)	0.77	0.77

Supplementary Table 2: Cryo-EM data collection, refinement and validation statistics for unliganded CD163

	Unliganded dimeric CD163	Unliganded trimer with disordered arms	Unliganded trimer with arm contacts
**Data collection and processing**			
Microscope		Titan Krios	
Detector		Gatan K3 with BioQuantum Imaging Filter	
Magnification		58,149	
Voltage (kV)		300	
Electron exposure (e-/Å^2)		42.82	
Defocus range (μm)		-0.6 to -1.8	
Pixel size (Å)		0.832	
Symmetry imposed		C1	
Initial particle images (no.)		13,772,487	
Final particle images (no.)	723,620	81,871	432,423
Map resolution (Å)	3.10	3.81	3.37
FSC threshold 0.143			
Map resolution range (Å)	2.57 – 40.5	3.08 – 60.8	1.78 – 51.6
FSC threshold 0.5			
**Refinement**			
Model resolution (Å)	3.31		
FSC threshold 0.5			
Model composition			
Non-hydrogen atoms	10616		
Protein residues	1382		
Ligands	18		
B factors (Å^2)			
Protein	91.71		
Ligands	109.73		
R.m.s deviations			
Bond lengths (Å)	0.003		
Bond angles (°)	0.646		
Validation			
MolProbity score	1.78		
Clashscore	8.09		
Poor rotamers (%)	0.18		
Ramachandran plot			
Favoured (%)	95.0		
Allowed (%)	5.0		
Disallowed (%)			
Model vs. data fit			
CC (mask)	0.78		
CC (box)	0.74		

Supplementary Table 3: interactions table

CD163			Liganded dimer		Liganded trimer		Unliganded dimer	
CD163 residue	Residue part	Interaction	Ligand residue	Residue part	Ligand residue	Residue part	$CD163_B$ residue	Residue part
D185(A)	OD1/OD2	Ca^{2+} coordination	Hp(K262)	NZ	Hp(K262)	NZ		
D186(A)	OD1/OD2	Ca^{2+} coordination	Hp(K262)	NZ	Hp(K262)	NZ		
S212(A)	OG	Hydrogen bond					W350	O
S212(A)	O	Hydrogen bond					K352	NZ
N213 (A)	OD1	Hydrogen bond					H353	N
E216(A)	OE1/OE2	Salt bridge	Hp(R252)	NH1	Hp(R252)	NH1/NH2		
D224(A)	OD1/OD2	Ca^{2+} coordination	Hp(K262)	NZ	Hp(K262)	NZ		
D225(A)	OD1/OD2	Ca^{2+} coordination	Hp(K262)	NZ	Hp(K262)	NZ		
N247(A)	OD1	Ca^{2+} coordination	Hp(K262)	NZ	Hp(K262)	NZ		
N247(A)	ND2	Hydrogen bond	Hp(E261)	OE2	Hp(E261)	OE1		
D249(A)	OD2	Salt bridge	Hp(R252)	NE	Hp(R252)	NE		
D249(A)	OD1	Hydrogen bond	Hp(S257)	OG	Hp(S257)	OG		
D249(A)	OD2	Hydrogen bond	Hp(T258)	N/OG1	Hp(T258)	N/OG1		
H250 (A)	NE2	Salt bridge					E286	OE2
E252(A)	OD1/OD2	Ca^{2+} coordination	Hp(K262)	NZ	Hp(K262)	NZ		
E252(A)	OD1/OD2	Hydrogen bond					Q284	NE2
Q284(A)	OD1/ND2	Hydrogen bond					H353	ND1/NE2
D292(A)	OD1/OD2	Ca^{2+} coordination	Hbα(K12)	NZ	Hbα(K12)	NZ		
D293(A)	OD1/OD2	Ca^{2+} coordination	Hbα(K12)	NZ	Hbα(K12)	NZ		
D293(B)	OD1/OD2	Salt bridge	Hbβ(K83)	NZ	Hbβ(K67)	NZ		
D293(B)	OD1/OD2	Hydrogen bond			Heme of Hbβ	O1A/O2A		
D293(C)	OD1/OD2	Salt bridge			Hbα(K91)	NZ		
S332(B)	OG	Hydrogen bond	Hbβ(E91)	OE1/OE2				
K346(C)	NZ	Hydrogen bond			Hp(N148)	OD1		
H347(C)	O	Hydrogen bond			Hp(S150)	N		
K352(A)	NZ	Salt bridge					D293	OD1/2
K352(A)	NZ	Salt bridge					E359	OE1/2
H353(B)	O	Hydrogen bond			Hbβ(K96)	NZ		
Y354(A)	Side chain	Hydrophobic, H-bond	Hbα(K8)	Side chain	Hbα(K8)	Side chain		
Y354(A)	OH	Hydrogen bond	Hbα(D75)	OD1/OD2	Hbα(D75)	OD2	N356/N358	OD1
Y354(B)	OH	Hydrogen bond	Hp(K168/K111)	NZ				
Y354(B)	Side chain	Hydrophobic			Hbβ(L97)	Side chain		
Y354(C)	OH	Hydrogen bond			Hbα(H90 and D86)	ND1 or OD1/OD2		
H357(A)	ND1/NE2	Hydrogen bond					H464	ND1/NE2
E359(A)	OD1/OD2	Ca^{2+} coordination	Hbα(K12)	NZ	Hbα(K12)	NZ		

E359(C)	OE2	Salt bridge			Hbα(K91)	NZ
R400(B)	NH1/NH2	Hydrogen bond	Hbβ(N81)	OD1/ND2		
R400(C)	NH1/NH2	Hydrogen bond			Hbα(P45)	O
G401(B)	H	Hydrogen bond	Hbβ(D80)	OD2		
S441(B)	OG	Hydrogen bond	Hbβ(H78)	NE2		
T461(A)	OG1	Hydrogen bond			Hbα(K17)	NZ
D463(A)	OD1/OD2	Salt bridge			Hbα(K17)	NZ
D463(B)	OD1/OD2	Salt bridge			Hbβ(H78)	NE2
D463(C)	OD1/OD2	Salt bridge			Hbα(H46)	ND1/NE2
H464(A)	ND1/NE2	Hydrogen bond	Hbα(H21)	ND1/NE2		
H464(B)	ND1	Hydrogen bond			Hbβ(A77)	O
Y465(B)	OH	Hydrogen bond	Hbβ(H3)	ND1	Hbβ(D74)	OD1
Y465(C)	OH	Hydrogen bond			Hbα(H46)	ND1/NE2

Supplementary Table 4: Measurement of binding constants

SPR was used to measure K_D and kinetic constants for the binding of WT and R809T CD163 to Hp(1-1)Hb, Hp(2-2)Hb and HpSPHb. The kinetic parameters were individually quantified using BIAevaluation (Cytiva) for every replicate, which were used to calculate the mean ± S.E.M. provided here. MST was used to determine the K_D values for binding of CD163 to Hb and Hp(1-1). These were derived from a fit to a curve for the combined triplicate, plotted as ΔFnorm. n = 3 for every interaction listed.

Method	CD163	Ligand	K_D ± S.E.M. (nM)	k_a ± S.E.M. (M^{-1} s^{-1})	k_d ± S.E.M. (s^{-1})
SPR	WT	Hp(1-1)Hb	0.276 ± 0.025	(6.16 ± 0.37) x 10^5	(1.68 ± 0.07) x 10^{-4}
	WT	Hp(2-2)Hb	0.158 ± 0.157	(4.05 ± 0.63) x 10^5	(4.41 ± 4.38) x 10^{-5}
	WT	HpSPHb	1.29 ± 0.09	(6.95 ± 0.65) x 10^5	(8.88 ± 0.29) x 10^{-4}
	R809T	Hp(1-1)Hb	1.21 ± 0.02	(1.05 ± 0.07) x 10^7	(1.26 ± 0.06) x 10^{-2}
	R809T	Hp(2-2)Hb	0.0311 ± 0.0177	(2.48 ± 0.86) x 10^6	(7.33 ± 3.83) x 10^{-5}
	R809T	HpSPHb	38.4 ± 17.4	(1.84 ± 0.66) x 10^6	(9.13 ± 4.45) x 10^{-2}
MST	WT	Hb	0.185 ± 0.026 μM		
	WT	Hp	26.6 ± 27.6 μM		
	R809T	Hb	1.87 ± 1.35 μM		
