# Supplementary material for: Scavenger receptor CD163 multimerises to allow uptake of diverse ligands
Source: Nat Commun. 2025 Jul 18;16:6623. doi: 10.1038/s41467-025-62054-9 (PMC12274614; doi:10.1038/s41467-025-62054-9)
Supplement: Supplementary file 2 — Reporting Summary [file 41467_2025_62054_MOESM2_ESM.pdf]

nature portfolio

Corresponding author(s): Matt Higgins

Last updated by author(s): 22-6-2025

Reporting Summary

Nature Portfolio wishes to improve the reproducibility of the work that we publish. This form provides structure for consistency and transparency in reporting. For further information on Nature Portfolio policies, see our Editorial Policies and the Editorial Policy Checklist.

Statistics

For all statistical analyses, confirm that the following items are present in the figure legend, table legend, main text, or Methods section.

n/a	Confirmed	
☐	☒	The exact sample size (*n*) for each experimental group/condition, given as a discrete number and unit of measurement
☐	☒	A statement on whether measurements were taken from distinct samples or whether the same sample was measured repeatedly
☒	☐	The statistical test(s) used AND whether they are one- or two-sided<br>*Only common tests should be described solely by name; describe more complex techniques in the Methods section.*
☒	☐	A description of all covariates tested
☒	☐	A description of any assumptions or corrections, such as tests of normality and adjustment for multiple comparisons
☐	☒	A full description of the statistical parameters including central tendency (e.g. means) or other basic estimates (e.g. regression coefficient) AND variation (e.g. standard deviation) or associated estimates of uncertainty (e.g. confidence intervals)
☒	☐	For null hypothesis testing, the test statistic (e.g. *F*, *t*, *r*) with confidence intervals, effect sizes, degrees of freedom and *P* value noted<br>*Give P values as exact values whenever suitable.*
☒	☐	For Bayesian analysis, information on the choice of priors and Markov chain Monte Carlo settings
☒	☐	For hierarchical and complex designs, identification of the appropriate level for tests and full reporting of outcomes
☒	☐	Estimates of effect sizes (e.g. Cohen's *d*, Pearson's *r*), indicating how they were calculated

Our web collection on statistics for biologists contains articles on many of the points above.

Software and code

Policy information about availability of computer code

Data collection	Software used for data collection are commercially available or openly accessible. Cryo-EM data were collected using EPU version 2.9.0.1519 (Thermo Fisher). SPR data were acquired using T200 Biacore Software version 2.0 (GE Healthcare). FACSDiva v9.2 (BD Biosciences) was employed to collect flow cytometry data. MST data were acquired using NT Control v2.0.2.29 (NanoTemper).
Data analysis	Data analysis was performed as described in the methods section using commercially available or openly accessible software. Software used for cryo-EM data processing (SIMPLE 3.0, CryoSPARC v4.2-4.5 and DeepEMhancer v1.0.0) are standard and freely available to academic users. Model building and refinement was performed with Coot version 0.9.8.8, ISOLDE v1.5 and PHENIX v1.20.1-4487. AlphaFold2-predicted human CD163 used as a starting model for model building was downloaded from the AlphaFold Protein Structure Database, with the model created with AlphaFold Monomer v2.0. ChimeraX v1.7 was used for structure visualisation. BIAevaluation software v1.0 is provided with the Biacore T200 SPR system and is standard in the field. Flow cytometry data were analysed using FlowJo v10.10. GraphPad Prism v10.3.1 was used to generate graphs, fit MST curves and derive IC50 values from FlowJo-quantified flow cytometry data. Adobe Illustrator was used to generate figures.

For manuscripts utilizing custom algorithms or software that are central to the research but not yet described in published literature, software must be made available to editors and reviewers. We strongly encourage code deposition in a community repository (e.g. GitHub). See the Nature Portfolio guidelines for submitting code & software for further information.

Data

Policy information about availability of data

All manuscripts must include a data availability statement. This statement should provide the following information, where applicable:

- Accession codes, unique identifiers, or web links for publicly available datasets
- A description of any restrictions on data availability
- For clinical datasets or third party data, please ensure that the statement adheres to our policy

Cryo-EM maps are available from the Electron Microscopy Data Bank under accession codes EMD-52078 (Dimeric CD163:HpHb), EMD-52079 (Trimeric CD163:HpHb), EMD-52080 (Unliganded dimeric CD163 with arm-arm contacts), EMD-52081 (Unliganded trimeric CD163 with arm-arm contacts) and EMD-52082 (Unliganded trimeric CD163 with disordered arms). Coordinates are available from the Protein Data Bank under accession codes 9HEJ (Dimeric CD163:HpHb), 9HEK (Trimeric CD163:HpHb) and 9HEL (Unliganded dimeric CD163 with arm-arm contacts). Uncropped gels and source data for all graphs generated in this study are provided in "Source Data.xlsx".

Research involving human participants, their data, or biological material

Policy information about studies with human participants or human data. See also policy information about sex, gender (identity/presentation), and sexual orientation and race, ethnicity and racism.

Reporting on sex and gender	N/A
Reporting on race, ethnicity, or other socially relevant groupings	N/A
Population characteristics	N/A
Recruitment	N/A
Ethics oversight	N/A

Note that full information on the approval of the study protocol must also be provided in the manuscript.

Field-specific reporting

Please select the one below that is the best fit for your research. If you are not sure, read the appropriate sections before making your selection.

☒ Life sciences ☐ Behavioural & social sciences ☐ Ecological, evolutionary & environmental sciences

For a reference copy of the document with all sections, see nature.com/documents/nr-reporting-summary-flat.pdf

Life sciences study design

All studies must disclose on these points even when the disclosure is negative.

Sample size	Sample sizes are described in figure legends and methods. No statistical method was used to predetermine sample size. Experiments were planned based on previous studies using equivalent methods and scoping experiments. Statistical tests were used to assess whether sample sizes were sufficient.
Data exclusions	For cryo-EM workflows particles not contributing to classes of interest were excluded from the final reconstructions as standard.
Replication	The number of repeats for each relevant experiment are given in figure legends and the methods. Typically, experiments were performed in independent technical triplicates. Cell-based uptake assays were conducted in biological triplicate.
Randomization	As is standard for cryo-EM workflows, particles were split randomly into two halves and refined separately, as is automatically implemented in CryoSPARC. Randomisation of other experiments was not relevant to this study as no subjective judgements were required about which data to include, exclude, or measure.
Blinding	The investigators were not blinded to the group allocation during the experiment and/or when assessing the outcome, as analysis were performed on quantitative endpoints that are not subject to investigator bias.

Reporting for specific materials, systems and methods

We require information from authors about some types of materials, experimental systems and methods used in many studies. Here, indicate whether each material, system or method listed is relevant to your study. If you are not sure if a list item applies to your research, read the appropriate section before selecting a response.

Materials & experimental systems

n/a	Involved in the study
[x]	[ ] Antibodies
[ ]	[x] Eukaryotic cell lines
[x]	[ ] Palaeontology and archaeology
[x]	[ ] Animals and other organisms
[x]	[ ] Clinical data
[x]	[ ] Dual use research of concern
[x]	[ ] Plants

Methods

n/a	Involved in the study
[x]	[ ] ChIP-seq
[ ]	[x] Flow cytometry
[x]	[ ] MRI-based neuroimaging

Eukaryotic cell lines

Policy information about cell lines and Sex and Gender in Research

Cell line source(s)	Commercially available Expi293F™ cells (ThermoFisher, cat: A14635) were used for recombinant protein expression. Commercially available Flp-In™-293 cells (ThermoFisher cat: R78007) were used to generate stably transfected, CD163-expressing cells for uptake assays.
Authentication	The cells were freshly obtained from commercial sources and were not validated.
Mycoplasma contamination	The cells were freshly obtained from commercial sources and were not validated.
Commonly misidentified lines (See ICLAC register)	N/A

Plants

Seed stocks	N/A
Novel plant genotypes	N/A
Authentication	N/A

Flow Cytometry

Plots

Confirm that:

- [x] The axis labels state the marker and fluorochrome used (e.g. CD4-FITC).
- [x] The axis scales are clearly visible. Include numbers along axes only for bottom left plot of group (a 'group' is an analysis of identical markers).
- [x] All plots are contour plots with outliers or pseudocolor plots.
- [x] A numerical value for number of cells or percentage (with statistics) is provided.

Methodology

Sample preparation	Stably transfected or untransfected Flp-In™-293 cells were washed in PBS and subjected to DMEM with 25 mM HEPES, fluorescently labelled ligand and optionally competitor ligand and 2.0 mM EGTA. After a 30 min incubation at 37 °C, 5 % CO2, cells were washed in PBS, trypsinised and washed again. Following addition of DRAQ7 (Abcam) to 3000 nM, cells were subjected to FACS measurement.
Instrument	LSRFortessa X-20 Cell Analyzer (BD Biosciences)
Software	To collect data, BD FACSDiva Software v9.2 was used. Data were subsequently analysed using FlowJo v10.10.
Cell population abundance	N/A
Gating strategy	Gating was only used to obtain single, live cells but not for analysis of ligand uptake.<br>Gating was achieved by plots of forward scatter area against side scatter area (gate = P1). Doublet discrimination required

gating of forward scatter width against forward scatter height (gate = P2). Live-dead cell discrimination was achieved by plotting forward scatter area against the intensity on 561 780-60-A (gate = P3), which detects DRAQ7-stained cells. This gating strategy was consistently applied on every sample in this study to generate histograms of single, live cells plotted on 561 610-20-A, detecting internalised Alexa Fluor 594-labelled ligands. The mean fluorescence intensity of the populations in these histograms was calculated using FlowJo v10.10 to quantify ligand uptake.

☒ Tick this box to confirm that a figure exemplifying the gating strategy is provided in the Supplementary Information.
